# Acupuncture for restless legs syndrome: A systematic review and meta-analysis protocol

**DOI:** 10.1097/MD.0000000000018902

**Published:** 2020-01-17

**Authors:** Kaiyu Huang, Shuang Liang, Dong Han, Rubao Guo, Lei Chen, Antoine Grellet

**Affiliations:** a Department of Acupuncture, Ningbo Municipal Hospital of TCM, Affiliated Hospital of Zhejiang Chinese Medical University, Ningbo; b Center for Diagnosis and Treatment of Intervertebral Disc Disease, The Third Affiliated Hospital of Zhejiang Chinese Medical University, Hangzhou; c The First Clinical Medical College, Nanjing University of Chinese Medicine, Nanjing, China.

**Keywords:** acupuncture, meta-analysis, protocol, restless legs syndrome, systematic review

## Abstract

**Background:**

Restless legs syndrome (RLS) is a neurological disorder that causes an irresistible urge to move the legs. An increasing number of studies have been published in recent years to support the effectiveness of acupuncture for RLS. We will conduct a comprehensive systematic review and meta-analysis to evaluate the evidence of randomized controlled trials for acupuncture treatment of RLS.

**Methods:**

The following electronic databases will be searched: PubMed, Embase, Web of Science, Cochrane Library, the China National Knowledge Infrastructure, Chinese Biomedical Literature Database, and China Science and Technology Journal Database. The range of publication time will be from the inception of the database to September 2019. Two reviewers will independently conduct article selection, data collection, and risk of bias evaluation. Any disagreement will be resolved by discussion with the third reviewer. Review Manager Software 5.3 will be used for meta-analysis. The Cochrane risk of bias tool will be used to assess the risk of bias.

**Results:**

This study will provide a high-quality evidence to assess the effectiveness and safety of acupuncture for RLS.

**Conclusions:**

This systematic review will explore whether acupuncture is an effective and safe intervention for RLS.

**Registration:**

PROSPERO CRD42019148948.

## 1 Introduction

Restless legs syndrome (RLS), also called Willis–Ekbom disease, is a neurological disorder that causes an irresistible urge to move the legs.^[1]^ The symptoms can improve observably after moving the legs, and are worse in the evening.^[2]^ RLS can significantly disrupt patients’ sleep every night, reduce quality of life, and affect health status.^[3–5]^ RLS is also associated with the higher risk or prevalence of anxiety and depression and troubling insomnia.^[6]^ Up to 5% to 15% of adults suffer from RLS, and about 2.7% experience moderate to severe RLS.^[2,7]^ The prevalence rate in women is higher than that in men.^[8]^ Pramipexole, ropinirole, rotigotine, and gabapentin enacarbil are common drugs approved by the US Food and Drug Administration.^[9]^ Side effects such as sleepiness and dizziness are inevitable. Some medications are related with a progressive loss of response.^[10,11]^

Acupuncture, as a treatment means of traditional Chinese medicine (TCM), has been widely used for RLS management in Asian countries such as China, Japan, and Korea. An increasing number of studies have been published in recent years to support the effectiveness of acupuncture for RLS.^[12–14]^ Up to now, 1 previous systematic review assessing the effects of acupuncture for RLS has been published.^[15]^ Only 2 trials were included, and meta-analysis was not performed. From the point of view of evidence-based medicine, the effectiveness and safety of acupuncture for RLS is still inconclusive. The literature search was performed up to February 2007. Some new randomized controlled trials (RCTs) of acupuncture for RLS have been published. In view of this, we have an opportunity to re-evaluate the effectiveness and safety of acupuncture for RLS. Therefore, we will conduct a comprehensive systematic review and meta-analysis to evaluate the evidence of RCTs for acupuncture treatment of RLS.

## 2 Methods

### 2.1 Study registration

This study has been registered on PROSPERO (CRD42019148948). This meta-analysis will be performed following the reporting guidelines and criteria set in preferred reporting items for systematic reviews and meta-analyses (PRISMA) statement checklist.^[16]^

### 2.2 Eligibility criteria for study selection

#### 2.2.1 Types of studies

All RCTs of acupuncture for RLS will be considered for inclusion without language limitation. Cross-trials, animal experiments, and repeatedly published studies will be excluded.

#### 2.2.2 Types of participants

Participants who meet the diagnostic criteria of RLS will be included without restrictions of age, gender, and race.

#### 2.2.3 Types of interventions

Acupuncture therapy will be limited to manual acupuncture, electroacupuncture, and warm needle acupuncture. Studies including ear acupressure, acupuncture point injection, or laser acupuncture will be excluded. Studies with different acupuncture methods on acupuncture points selection will be excluded, such as body acupuncture against scalp acupuncture.

RCTs that have control groups with conventional treatments (such as drug therapy and physical therapy), sham acupuncture or no treatment will be included.

#### 2.2.4 Types of outcomes

Unpleasant sensations of RLS, as assessed using a visual analog scale, will be designated as the primary outcome. Secondary outcomes will include the International Restless Legs Syndrome Rating Scale, Pittsburgh Sleep Quality Index, and adverse events.

### 2.3 Search strategy

The following electronic databases will be searched: PubMed, Embase, Web of Science, Cochrane Library, the China National Knowledge Infrastructure, Chinese Biomedical Literature Database, and China Science and Technology Journal Database. The range of publication time will be from the inception of the database to September 2019. The detailed search strategy for PubMed is shown in Table 1. Identical search strategies will be used for other electronic databases.

**Table 1:**
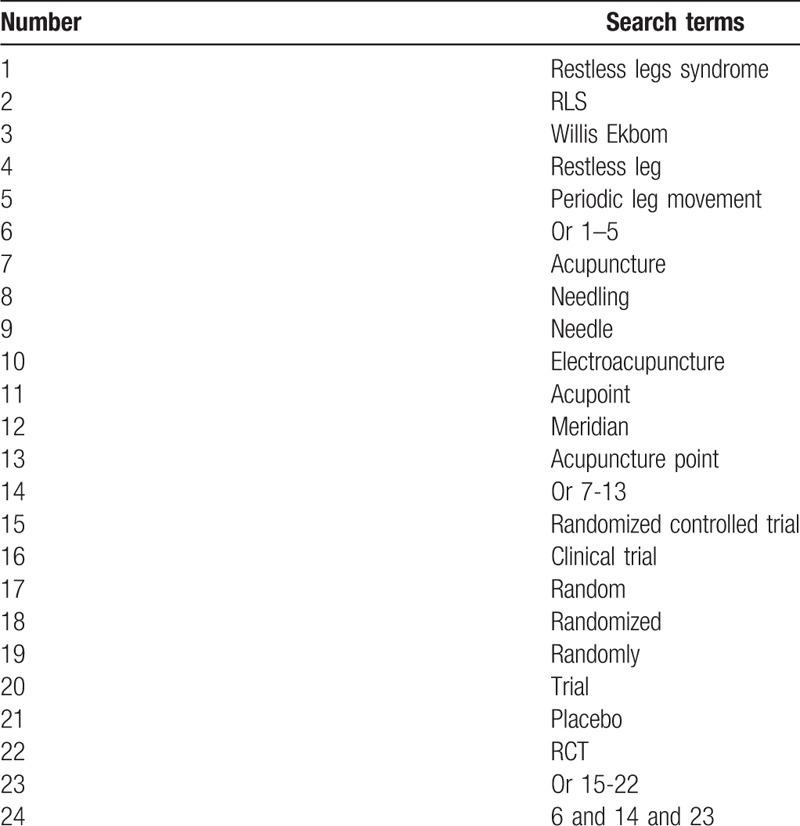
Search strategy for PubMed.

### 2.4 Selection of studies

After removing duplicates, 2 reviewers will independently evaluate all the eligible articles for inclusion. Titles and abstracts will be scanned to eliminate all irrelevant records. The remaining records will be read by full texts in further assessing the inclusion of the study. Any disagreement will be resolved by discussion with the third reviewer. A PRISMA flow chart will be designed to illustrate the study selection process.

### 2.5 Data extraction and management

After selection, 2 reviewers will independently conduct data extraction. Any disagreement will be resolved by discussion with the third reviewer. The general information will be extracted, including first author's name, year of publication, title of journal, study design, patient information, experimental intervention, control intervention, duration of intervention, and outcomes. If some important information is missing, we will contact original authors by email to request detailed information about the research.

### 2.6 Assessment of risk of bias

In this study, the Cochrane risk of bias assessment tool will be used to assess the risk of bias of the selected studies. It consists 7 items: random sequence generation, allocation concealment, blinding of participants and personnel, blinding of outcome assessment, incomplete outcome data, selective reporting, and other bias. A bias value of “high,” “unclear,” or “low” was given for each item. These 7 items were assessed independently by 2 reviews. The rating results will be cross-checked and the difference will be solved by the third reviewer.

### 2.7 Data synthesis and analysis

#### 2.7.1 Measurement of treatment effect

Continuous outcomes will be presented as mean difference or standardized mean difference with 95% confidence interval. Risk ratio will be used for dichotomous outcomes with 95% confidence interval.

#### 2.7.2 Assessment of heterogeneity

Heterogeneity will be examined using the *I*^*2*^ test. The *I*^*2*^ value >50% means significant heterogeneity, and the random effects model will be used. Otherwise, the *I*^*2*^ value ≤50% means minor heterogeneity, and the fixed effects model will be utilized.

#### 2.7.3 Data synthesis

Review Manager Software 5.3 will be used for data synthesis. The random effects model or fixed effects model will be selected according to the *I*^*2*^ value. All data will be analyzed with 95% confidence interval. If significant heterogeneity still exists after subgroup analysis, meta-analysis will not be pooled, and descriptive summary will be reported.

#### 2.7.4 Subgroup analysis

If significant heterogeneity is found, subgroup analysis will be performed according to different characteristics, treatment methods, and outcome measurements.

#### 2.7.5 Sensitivity analysis

Sensitivity analysis will be conducted to check the robustness and reliability of pooled outcome results by excluding low-quality studies and small studies.

#### 2.7.6 Reporting bias

Publication bias will be assessed with funnel plot and Egger regression analysis if sufficient trials (≥10 trials) are included.

## 3 Discussion

Irresistible urge to move the legs is the key clinical feature of RLS. That develops at night and can be more serious as the night progress.^[17]^ Moving the legs or walking improves the urge. Hence, RLS interferes with rest and sleep, leading to poor quality of life and productivity.^[18]^ Acupuncture appears to be beneficial in improving RLS severity and sleep in some people with RLS.^[19]^ The previous systematic review did not provide sufficient evidence to support the hypotheses that acupuncture is more effective for RLS than no treatment or other therapies.^[15]^ The literature search was performed up to February 2007 and only 2 trials with 170 patients were included. Meta-analysis and funnel plot were not reported. Therefore, we will comprehensively search to include recent studies to provide better evidence for the treatment of RLS.

We believe that this systematic review will provide clinical evidence for the efficacy and safety of acupuncture treatment of RLS, inform our understanding of the value of acupuncture in treating RLS, and provide helpful evidence for treating RLS by applying acupuncture in clinical practice.

## Author contributions

**Conceptualization:** Kaiyu Huang, Shuang Liang, Lei Chen.

**Data curation:** Dong Han, Rubao Guo.

**Formal analysis:** Shuang Liang, Dong Han, Rubao Guo, Lei Chen.

**Investigation:** Dong Han, Antoine Grellet.

**Methodology:** Kaiyu Huang, Lei Chen.

**Project administration:** Lei Chen.

**Resources:** Kaiyu Huang, Lei Chen, Rubao Guo.

**Software:** Kaiyu Huang, Shuang Liang, Dong Han.

**Supervision:** Kaiyu Huang, Shuang Liang, Dong Han.

**Validation:** Rubao Guo, Lei Chen.

**Visualization:** Kaiyu Huang, Shuang Liang.

**Writing – original draft:** Kaiyu Huang, Shuang Liang.

**Writing – review and editing:** Lei Chen, Antoine Grellet.
